# Social inequalities in health: measuring the contribution of housing deprivation and social interactions for Spain

**DOI:** 10.1186/1475-9276-11-77

**Published:** 2012-12-14

**Authors:** Rosa M Urbanos-Garrido

**Affiliations:** 1 Department of Public Finance, School of Economics, Complutense University of Madrid, Campus de Somosaguas s/n, 28223, Pozuelo de Alarcón, Spain

**Keywords:** Health inequalities, Equity, Housing deprivation, Social interactions, Social exclusion, Spain

## Abstract

**Introduction:**

Social factors have been proved to be main determinants of individuals’ health. Recent studies have also analyzed the contribution of some of those factors, such as education and job status, to socioeconomic inequalities in health. The aim of this paper is to provide new evidence about the factors driving socioeconomic inequalities in health for the Spanish population by including housing deprivation and social interactions as health determinants.

**Methods:**

Cross-sectional study based on the Spanish sample of European Statistics on Income and Living Conditions (EU-SILC) for 2006. The concentration index measuring income-related inequality in health is decomposed into the contribution of each determinant. Several models are estimated to test the influence of different regressors for three proxies of ill-health.

**Results:**

Health inequality favouring the better-off is observed in the distribution of self-assessed health, presence of chronic diseases and presence of limiting conditions. Inequality is mainly explained, besides age, by social factors such as labour status and financial deprivation. Housing deprivation contributes to pro-rich inequality in a percentage ranging from 7.17% to 13.85%, and social interactions from 6.16% to 10.19%. The contribution of some groups of determinants significantly differs depending on the ill-health variable used.

**Conclusions:**

Health inequalities can be mostly reduced or shaped by policy, as they are mainly explained by social determinants such as labour status, education and other socioeconomic conditions. The major role played on health inequality by variables taking part in social exclusion points to the need to focus on the most vulnerable groups.

**JEL Codes:**

H51, I14, I18

## Introduction

Social inequalities in health have been widely studied across countries [1-12]. New evidence about the impact of structural factors on health and health inequalities has also grown over recent years [6-12]. Empirical studies not only focus on quantifying the socioeconomic health gradient, but in many cases provide guidance for public action aimed at fighting this problem [5,10,12-16], which is one of the main concerns of public sectors in the European Union [17,18]. The reduction of social health inequalities also ranks high in Spain, where basic health legislation explicitly includes it as a goal [19-21]. Moreover, the Ministry of Health in 2008 launched a national commission to address this issue. In 2010 the commission presented a report with its findings and a set of recommendations about the strategic actions to be promoted [16]. These recommendations have been grouped into four clusters which together make up the National Strategy of Equity in Health: the development of information systems on health equity, the promotion of multi-sectorial tools moving towards the concept of ‘Health and Equity in All Policies’, and the development of both a comprehensive plan to support child and youth health and a plan of political visibility for the strategy. During the Spanish EU Presidency in the first half of 2010, health equity was also placed very high on the agenda [15].

Previous studies have explored the impact on health inequality of what the Commission on Social Determinants of Health [10] considers in its conceptual framework as structural factors, such as education, income and employment [6,8-10,22,23]. Within this framework, other sets of variables are seen as intermediate health determinants with impact on the distribution of health and well-being, including material circumstances, social cohesion or psychosocial factors. Some of the variables used to approach these factors have not been analysed until now, as happens with housing deprivation and social interactions. Even though their impact on health has been studied [24-29], their effect on socioeconomic health inequalities remains unknown.

Moreover, both factors may be considered as main elements of the social integration process. At the moment, little empirical research has explicitly focused on the relationship between social exclusion and health inequalities [30]. This paper provides new evidence about that relationship by exploring the impact of housing deprivation and social interactions on Spanish health inequalities, thanks to the information provided by the European Statistics on Income and Living Conditions (EU-SILC). Several proxies of ill-health are used to test how the contribution of health determinants varies across models.

## Methods and data

Social inequalities in health may be measured by using different methods, such as the rate ratio between two extreme groups, the Relative Index of Inequality, the Slope Index of Inequality, the Population-Attributable Risk or the Index of Dissimilarity, among others [31,32]. However, the most common instruments for measuring inequalities (and inequity) in health and health care use are concentration indices, which capture the socioeconomic dimension of inequalities, take into account the whole population, are sensitive to changes in population distribution along the socioeconomic scale, offer the possibility of visual representation through the concentration curve and allow testing dominance relationships [32].

The concentration curve represents the relationship between the cumulative proportion of population ranked by income and the cumulative proportion of ill-health. Moreover, the concentration index *(C)* is calculated as twice the area between the concentration curve and the 45º line. A negative index shows that ill-health is concentrated in individuals with relatively low income and is represented by a concentration curve above the 45º line.

If ill-health is represented by the variable *y*, the concentration index *C* is typically expressed by (1):

(1) $$C=\frac{2}{\bar{y}}\mathrm{cov}\left(y,r\right)$$

where − 1 ≤ *C* ≤ 1, being $\bar{y}$ the mean of *y*, and *r* the cumulative percentage that each individual represents over the total population once the latter has been ranked by income. Only when *C = 0* can it be concluded that there is no socioeconomic inequality in health. When the health variable is dichotomous, the concentration index has to be corrected in order to allow comparisons between groups of individuals that may present different levels of average health [33]. The adjusted concentration index *(E(y))* has been suggested by Erreygers [34] and can be calculated as follows:

(2) $$E\left(y\right)=\frac{4\bar{y}}{y^{\max}-y^{\min}}C$$

where *y*^*max*^ and *y*^*min*^ are the extremes of the health variable.

Kakwani et al. [35] propose the following formula to calculate the concentration index, which indicates if the index is statistically significant:

(3) $$2\sigma_{r}^{2}\left(\frac{y_{i}}{y}\right)=\alpha +\beta r_{i}+\varepsilon_{i}$$

where *σ*_*r*_^2^ is the variance of *r*, and the OLS estimator of *β* is the concentration index. In fact, the standard error of $\hat{\beta }$ is not exactly the same as that corresponding to the concentration index, given that the former does not consider the sample variability of $\bar{y}$. However, as the variation is not significant when variability is considered, equation (3) may be used to identify if the concentration index is or is not significant [36].

Wagstaff et al. [37] prove that the concentration index may be expressed as a weighted sum of the concentration indices for the explanatory factors of inequality. Thus, if there is a linear relationship between *y* and a set of k explanatory variables *x*, the concentration index can be expressed as (4):

(4) $$C=\sum_{k}\left(\frac{\beta_{k}{\bar{x}}_{k}}{\bar{y}}\right)\;C_{k}+\frac{GC_{\varepsilon }}{\bar{y}}$$

where ${\bar{x}}_{k}$ is the mean of the variable determining ill-health, *C*_*k*_ are the concentration indices for the k explanatory factors considered and *GC*_*ε*_ is the generalized concentration index for the error term, which reflects inequality in health not explained by the set of regressors. Therefore, the concentration index (*C*) may be decomposed into a weighted sum of concentration indices for the explanatory variables, where the weights are ill-health elasticity with respect to each of the *x*_*k*_ variables [38].

Equation (4) only applies if the relationship between the dependent variable and the regressors is linear. However, ill-health variables usually are estimated by using non-linear models, where health of the i individual is measured by a continuous, non-observed latent variable *y*_i_^*^, which is represented by a discrete and observable variable *y*_*i*_, so that:

(5) $$y_{i}=\{\begin{array}{c} 1\;\mathrm{if}\;y_{i}^{*}>0\\ 0\;\mathrm{if}\;y_{i}^{*}=0 \end{array}$$

In this case, decomposition analysis is possible only if some linear approximation to the non-linear model is made. This can be done by using estimates of the partial effects evaluated at the means [38]. Thus, *C* can be rewritten as:

(6) $$C=\underset{k}{\Sigma }\left(\frac{\beta_{k}^{m}{\bar{x}}_{k}}{\bar{y}}\right)C_{k}+\frac{\mathrm{GC\varepsilon}}{\bar{y}}$$

where *β*_*k*_^*m*^ are the partial effects (*dy*/*dx*_*k*_) evaluated at sample means, and *ε* is the error term, which now includes the approximation error.

Data used correspond to the Spanish sample of adults from Statistics on Income and Living Conditions (EU-SILC) 2006. EU-SILC provides multi-dimensional data on income, social exclusion and living conditions in the European Union, which are collected at the household level. Information about health and other characteristics such as labour status or education, in addition to demographic variables, refer to individuals aged 16 and over [39]. For the year 2006, EU-SILC includes a specific module on social participation, only available for that wave of the survey, which allows for testing the influence of social interactions on health inequalities. The final sample used consists of 25,498 individuals ^a^.

According to O’Donnell et al. [36], different health variables should be used to accurately analyze the distribution of health. EU-SILC provides three different measures of ill-health: self-assessed health, the presence of chronic diseases and the presence of conditions limiting daily activities. Self-assessed health is a subjective measure of health that may involve biases in the measurement of inequalities, as it may be systematically correlated with characteristics such as sex, age, income level or education [40]. However, it is a good proxy for other objective variables such as the mortality rate, and it also provides a wide picture of the overall health status of individuals. The presence of chronic diseases can be considered a measure of medical health, as it relates to the deviation of the ‘medical standards’ (presence of certain diseases, symptoms or disabilities) [32]. Finally, the presence of conditions limiting daily activities is a functional measure, as it relates to the inability to perform everyday tasks. Each of these measures therefore represents a different dimension of health. In this paper, inequality will be calculated and decomposed separately for these three variables. However, it should be noticed that all of them are self-reported, which may imply a potential reporting bias.

Dependent variables are defined as follows. First, self-assessed health (*SAH*) will take value one if the individual declares his/her health as fair, poor or very poor, and zero if health is perceived as good or very good. This categorization has been used in previous studies [2,12,36]. Second, the presence of chronic conditions is represented by a dummy (*chronic*) which takes value one if the individual declares any chronic disease, disability or condition. Third, the variable representing the functional dimension of health (*limit*) takes value one if the individual declares any kind of limitations in daily activity (intense or not) due to health problems in the preceding six months (zero otherwise). In the estimation of ill-health, probit models have been used where the error term is assumed to be distributed as a N (0,1).

The explanatory variables, which are described in Table 1, include a set of demographic and socioeconomic characteristics, along with regressors representing additional living conditions available in the EU-SILC and considered as relevant determinants of health, such as housing conditions, social interactions and the geographic environment [10].

**Table 1:** Descriptive statistics

**Variables**		**Definition**	**Mean**	**Std. Dev.**	**Concentration index**
*Ill-health*	*SAH*	Self-assessed health: 1 if fair, poor or very poor; 0 if good or very good	0.292	0.454	−0.150
	*Chronic*	Chronic conditions: 1 if any chronic disease, disability or condition	0.218	0.413	−0.097
	*Limit*	Limitations in daily activity: 1 if any kind of limitations in daily activity due to health problems in the preceding six months	0.203	0.402	−0.101
*Age*					
	*Age1*	Age 16–34 (reference category)	0.326	0.469	0.045
	*Age2*	Age 35-44	0.207	0.405	0.034
	*Age3*	Age 45-49	0.239	0.426	0.055
	*Age4*	Age 60-74	0.148	0.355	−0.100
	*Age5*	Age > 75	0.080	0.271	−0.251
*Sex*					
	*Female*	Female	0.506	0.500	−0.023
	*Foreign*	Born outside Spain	0.050	0.219	−0.149
	*Couple*	Married or living with his/her partner	0.637	0.481	0.021
*Education*					
	*Ed1*	Primary education or below (reference category)	0.313	0.463	−0.233
	*Ed2*	Compulsory secondary education	0.229	0.420	−0.112
	*Ed3*	Non-compulsory and pre-university secondary education	0.206	0.405	0.071
	*Ed4*	Specific labour training	0.012	0.109	0.055
	*Ed5*	University graduate	0.240	0.427	0.346
*Labour status*					
	*Employed*	Employed in a full-time job (reference category)	0.481	0.590	0.180
	*Unemployed*	Unemployed	0.069	0.253	−0.252
	*Student*	Student or trainee	0.076	0.266	−0.098
	*Retired*	Retired	0.131	0.338	−0.140
	*Invalid*	Permanently disabled	0.018	0.134	−0.162
	*Home*	Engaged in housework or child care	0.124	0.329	−0.232
	*Otherinact*	Other inactive situations	0.043	0.204	−0.256
	*Part-time*	Employed in a part-time job	0.058	0.234	−0.010
*Social interactions*					
	*Freqfam1*	Daily or weekly face contacts with family members not belonging to the household (reference category)	0.620	0.485	−0.009
	*Freqfam2*	Family face contacts several times a month or once a month	0.229	0.420	0.063
	*Freqfam3*	Family face contacts less than once a month, or never	0.151	0.358	−0.058
	*Freqfriend1*	Daily or weekly face contacts with friends (reference category)	0.668	0.471	0.011
	*Freqfriend2*	Friends face contacts several times a month or once a month	0.208	0.406	0.066
	*Freqfriend3*	Friends face contacts less than once a month, or never	0.124	0.330	−0.171
	*Contfam1*	Daily or weekly non-face contacts with family (reference category)	0.680	0.466	0.037
	*Contfam2*	Family non-face contacts several times a month or once a month	0.213	0.409	−0.040
	*Contfam3*	Family non-face contacts less than once a month, or never	0.107	0.309	−0.158
	*Contfriend1*	Daily or weekly non-face contacts with friends (reference category)	0.595	0.491	0.073
	*Contfriend2*	Friends non-face contacts several times a month or once a month	0.224	0.417	−0.007
	*Contfriend3*	Friends non-face contacts less than once a month, or never	0.180	0.385	−0.233
	*Particip*	Participation in any organized non-work activity	0.655	0.475	0.025
*Degree of urbanization*					
	*Density1*	Very populated area (reference category)	0.529	0.499	0.105
	*Density2*	Medium populated area of residence	0.201	0.401	−0.055
	*Density3*	Sparsely populated area of residence	0.270	0.444	−0.164
*Housing deprivation*					
	*Dephousing1*	Home suffering from one deprivation problem	0.249	0.432	−0.089
	*Dephousing2*	Home suffering from two or more of deprivation problems	0.060	0.238	−0.393
*Financial deprivation*					
	*Findepriv1*	Suffers from one financial deprivation problem	0.194	0.395	−0.091
	*Findepriv2*	Suffers from two or more financial deprivation problems	0.278	0.448	−0.335
	*Nodentist*	Did not get dental treatment due to financial problems	0.032	0.175	−0.309
*Income*					
	*Eqhincome*	Log of equivalent household income	9.325	0.667	0.036
*Region of residence*					
	*Reg1*	Galicia	0.068	0.252	−0.119
	*Reg2*	Asturias	0.028	0.164	0.045
	*Reg3*	Cantabria	0.014	0.116	0.048
	*Reg4*	País Vasco	0.047	0.212	0.185
	*Reg5*	Navarra	0.015	0.120	0.256
	*Reg6*	Rioja	0.007	0.086	−0.024
	*Reg7*	Aragón	0.031	0.174	0.074
	*Reg8*	Madrid	0.133	0.340	0.152
	*Reg9*	Castilla y León	0.061	0.240	−0.118
	*Reg10*	Castilla-La Mancha	0.038	0.191	−0.121
	*Reg11*	Extremadura	0.024	0.153	−0.306
	*Reg12*	Cataluña	0.162	0.368	0.173
	*Reg13*	Comunidad Valenciana	0.109	0.312	−0.025
	*Reg14*	Islas Baleares	0.020	0.141	0.151
	*Reg15*	Andalucía	0.167	0.373	−0.166
	*Reg16*	Región de Murcia	0.029	0.167	−0.089
	*Reg17*	Ceuta	0.002	0.039	−0.139
	*Reg18*	Melilla	0.001	0.035	0.078
	*Reg19*	Islas Canarias	0.044	0.205	−0.164

Basic demographic characteristics are represented by age and sex. Other personal characteristics with potential influence on health include immigrant and family status. Socioeconomic indicators include educational level and labour status.

The variables representing social interactions indicate the frequency of contacts with family and friends. Face contacts with family members are represented by three dummies: *Freqfam1* indicates daily or weekly contacts with family members not belonging to the household (reference category); *Freqfam2* takes value one if the frequency of contacts is several times a month (but not weekly) or once a month; and *Frecfam3* indicates a lower frequency (at least once a year but less than once a month, or never). Similarly, the frequency of face contacts with friends is represented by the dummies *Freqfriend1* (reference category), *Freqfriend2* and *Freqfriend3*, and the frequency of non-face contacts with family and friends by dummies *Contfam1-Contfam3* and *Contfriend1-Contfriend3*, respectively (being *Contfam1* and *Contfriend1* the reference categories). An additional dummy (*Particip*) reflects if the individual participated in some kind of organized non-work activity in the preceding year (volunteering with political parties, professional, religious, recreational or sporting organizations, associations with charitable or humanitarian purposes or other kind of organizations). This set of variables could be considered as a proxy of individual social capital [27-29].

In order to proxy the housing conditions, two dummies based on the deprivation index used in the FOESSA report [41] are included in the analysis. *Dephousing1*, which equals 1 if the individual’s home shows one of the following deprivation problems: lack of toilet, lack of bath or shower, inability to maintain warm temperature during the winter, leaks, moisture or rot in floors, ceilings, foundations, windows or doors, or overcrowding; and *Dephousing2*, which equals one when the house has two or more of these problems (reference category: no deprivation).

Socioeconomic status is included in the model in two alternative ways. First, (the log of) equivalent household income (*Eqhincome*) is used. This variable is constructed from the annual net household income in the year preceding the interview together with information on household composition. The modified OECD equivalence scale was used to calculate equivalent income. Second, a set of variables representing financial deprivation is employed, based on information about the following household financial problems: inability to afford a meal with meat, chicken or fish at least every two days; inability to afford unexpected expenses or to pay for at least one holiday per week; lack of telephone, color TV, computer, washing machine or car (due to economic reasons); problems in making ends meet; or problems keeping the house at a comfortable temperature during the winter. Since this latter problem may be due either to lack of an adequate heating system or to limitation of its use for economic reasons, it has to be considered both as an element of housing and financial deprivation [41]. Thus, *Findepriv1* is a dummy taking value one when households suffer from one of the above-mentioned problems and *Findepriv2* another dummy taking value one when households suffer from two or more such problems (reference category: no financial deprivation). Additionally, a further variable is included: *Nodentist* is a dummy taking value one if the individual could not get dental treatment due to financial problems. This variable has been treated separately from other financial problems due to its direct relationship with health.

Finally, the geographic environment is proxied by the degree of urbanization of the place of residence and also by a set of regional dummies.

## Results and discussion

Table 1 provides descriptive statistics for all the variables considered in the analysis, and also the concentration indices which inform about inequality in the distribution of each variable. A negative (positive) sign indicates that the variable has a pro-poor (pro-rich) distribution. According to the data, 29.2% of the population declares his/her health as fair, poor or very poor, although the proportion of health problems is lower when alternative measures of ill-health are used. The percentage of people declaring chronic illnesses reach 21.8%, whilst 20.3% of the total sample suffers from limiting conditions.

Table 1 shows that a significant proportion of Spanish households suffer from some sort of housing or financial deprivation (30.9% and 47.2%, respectively). Although it is more frequent to suffer from only one type of housing problem, financial problems tend to accumulate. Variables representing social interactions show that the proportion of the population with a high frequency of face contacts with friends exceeds the percentage of people with high frequency of face contacts with family. The opposite happens when non-face contacts are considered. With respect to the attitude toward collective organizations, 65% of individuals participate in organized non-work activities.

The concentration indices show a significant inequality in the distribution of ill-health, which is relatively more concentrated among the poor. The values initially obtained were −0.129, -0.111 and −0.124 for self-assessed health, presence of chronic diseases and presence of limiting conditions, respectively. Once the correction proposed by Erreygers is applied, the adjusted indices become −0.150, -0.097 and −0.101, respectively. These values are consistent with previous literature [6,12].

Tables 2, 3, 4 report the total *C* decomposition for the models referring to self-assessed health, presence of chronic diseases and limiting conditions, respectively. Each table firstly shows the results obtained when the equivalent household income is used to proxy socio-economic status. The partial effects for the explanatory variables are followed by ill-health elasticity for each health determinant. Finally, the last two columns report, respectively, absolute and percentage contributions to total income-related inequality. The absolute contribution is the product of the elasticity and the concentration index for each factor, so it will depend both on the impact of each variable on health and on its unequal distribution by income. A negative absolute contribution implies that the variable contributes to inequality to the disadvantage of the poor, whereas a positive contribution indicates that inequality tends to favour the poor. The right-hand side of each table follows the same structure and refers to the results derived from the models using financial deprivation as a proxy of socio-economic status.

**Table 2:** Contributions to inequality in self-assessed health (n=25,498)

	**Income**				**Deprivation**			
**Variables**	**Partial effect**	**Elasticity**	**Contribution**	**% Contribution**	**Partial effect**	**Elasticity**	**Contribution**	**% Contribution**
*Age2*	0.124^***^	0.0878	0.0030	2.34%	0.126^***^	0.0897	0.0031	2.39%
*Age3*	0.254^***^	0.2079	0.0114	8.87%	0.260^***^	0.2130	0.0117	9.09%
*Age4*	0.367^***^	0.1860	−0.0187	−14.50%	0.381^***^	0.1930	−0.0194	−15.04%
*Age5*	0.503^***^	0.1376	−0.0345	−26.79%	0.519^***^	0.1421	−0.0356	−27.68%
*Female*	0.032^***^	0.0558	−0.0013	−0.97%	0.032^***^	0.0552	−0.0012	−0.97%
*Foreign*	0.012	0.0021	−0.0003	−0.24%	−0.004	−0.0007	0.0001	0.08%
*Couple*	−0.020^*^	−0.0439	−0.0009	−0.72%	−0.009	−0.0202	−0.0004	−0.33%
*Ed2*	−0.041^***^	−0.0321	0.0036	2.79%	−0.036^***^	−0.0279	0.0031	2.43%
*Ed3*	−0.062^***^	−0.0440	−0.0031	−2.44%	−0.046^***^	−0.0324	−0.0023	−1.80%
*Ed4*	−0.075^**^	−0.0031	−0.0002	−0.13%	−0.057	−0.0023	−0.0001	−0.10%
*Ed5*	−0.084^***^	−0.0688	−0.0238	−18.48%	−0.061^***^	−0.0500	−0.0173	−13.44%
*Unemployed*	0.069^***^	0.0162	−0.0041	−3.18%	0.051^***^	0.0121	−0.0031	−2.37%
*Student*	−0.110^***^	−0.0289	0.0028	2.19%	−0.106^***^	−0.0279	0.0027	2.12%
*Retired*	0.141^***^	0.0633	−0.0089	−6.89%	0.137^***^	0.0616	−0.0086	−6.71%
*Invalid*	0.630^***^	0.0394	−0.0064	−4.95%	0.623^***^	0.0389	−0.0063	−4.90%
*Home*	0.070^***^	0.0297	−0.0069	−5.36%	0.066^***^	0.0280	−0.0065	−5.05%
*Otherinact*	0.112^***^	0.0167	−0.0043	−3.31%	0.105^***^	0.0155	−0.0040	−3.09%
*Part-time*	0.005	0.0009	−0.0089	−0.01%	−0.002	−0.0004	0.0041	0.00%
*Freqfam2*	−0.014	−0.0112	−0.0007	−0.55%	−0.012	−0.0096	−0.0006	−0.47%
*Freqfam3*	0.018	0.0094	−0.0005	−0.42%	0.013	0.0066	−0.0004	−0.30%
*Freqfriend2*	0.020^*^	0.0141	0.0009	0.72%	0.017^*^	0.0124	0.0008	0.63%
*Freqfriend3*	0.088^***^	0.0374	−0.0064	−4.97%	0.080^***^	0.0340	−0.0058	−4.50%
*Contfam2*	- 0.014	−0.0103	0.0004	0.32%	−0.015	−0.0108	0.0004	0.33%
*Contfam3*	- 0.007	−0.0025	0.0004	0.30%	−0.009	−0.0033	0.0005	0.40%
*Contfriend2*	0.020^*^	0.0152	−0.0001	−0.09%	0.017	0.0128	−0.0001	−0.07%
*Contfriend3*	0.035^***^	0.0214	−0.0050	−3.88%	0.022^*^	0.0139	−0.0032	−2.51%
*Particip*	0.008	0.0187	0.0005	0.36%	0.008	0.0169	0.0004	0.32%
*Density2*	0.001	0.0009	0.0000	−0.04%	0.002	0.0016	−0.0001	−0.07%
*Density3*	0.006	0.0057	−0.0009	−0.73%	0.010	0.0089	−0.0015	−1.13%
*Dephousing1*	0.043^***^	0.0371	−0.0033	−2.56%	0.026^***^	0.0219	−0.0019	−1.51%
*Dephousing2*	0.170^***^	0.0352	−0.0138	−10.75%	0.112^***^	0.0231	−0.0091	−7.05%
*Eqhincome*	−0.007	−0.2336	−0.0084	−6.56%	-	-	-	-
*Findepriv1*	-	-	-	-	0.054^***^	0.0362	−0.0033	−2.57%
*Findepriv2*	-	-	-	-	0.110^***^	0.1048	−0.0351	−27.28%
*Nodentist*	-	-	-	-	0.100^***^	0.0109	−0.0034	−2.61%
*Reg2*	−0.071^***^	−0.0067	−0.0003	−0.24%	−0.058^***^	−0.0055	−0.0002	−0.19%
*Reg3*	−0.108^***^	−0.0051	−0.0002	−0.19%	−0.102^***^	−0.0048	−0.0002	−0.18%
*Reg4*	−0.087^***^	−0.0141	−0.0026	−2.02%	−0.073^***^	−0.0118	−0.0022	−1.69%
*Reg5*	−0.067^***^	−0.0034	−0.0009	−0.67%	−0.050^**^	−0.0025	−0.0006	−0.50%
*Reg6*	−0.073^***^	−0.0019	0.0000	0.04%	−0.062^***^	−0.0016	0.0000	0.03%
*Reg7*	−0.110^***^	−0.0118	−0.0009	−0.68%	−0.093^***^	−0.0100	−0.0007	−0.57%
*Reg8*	−0.090^***^	−0.0413	−0.0063	−4.86%	−0.081^***^	−0.0369	−0.0056	−4.36%
*Reg9*	−0.084^***^	−0.0177	0.0021	1.62%	−0.077^***^	−0.0162	0.0019	1.49%
*Reg10*	−0.114^***^	−0.0148	0.0018	1.40%	−0.106^***^	−0.0139	0.0017	1.31%
*Reg11*	−0.111^***^	−0.0091	0.0028	2.17%	−0.111^***^	−0.0091	0.0028	2.17%
*Reg12*	−0.105^***^	−0.0582	−0.0100	−7.81%	−0.096^***^	−0.0535	−0.0092	−7.17%
*Reg13*	−0.109^***^	−0.0408	0.0010	0.79%	−0.109^***^	−0.0406	0.0010	0.79%
*Reg14*	−0.101^***^	−0.0070	−0.0011	−0.82%	−0.096^***^	−0.0066	−0.0010	−0.78%
*Reg15*	−0.095^***^	−0.0544	0.0090	7.00%	−0.098^***^	−0.0560	0.0093	7.21%
*Reg16*	−0.042^**^	−0.0041	0.0004	0.29%	−0.041^**^	−0.0040	0.0004	0.28%
*Reg17*	−0.076^***^	−0.0004	0.0001	0.04%	−0.073^**^	−0.0004	0.0001	0.04%
*Reg18*	−0.060^*^	−0.0003	0.0000	−0.02%	−0.051	−0.0002	0.0000	−0.01%
*Reg19*	−0.087^***^	−0.0131	0.0022	1.68%	−0.096^***^	−0.0145	0.0024	1.85%
*Residual*			0.0038	2.91%			0.0181	14.04%
*Total*				−100.00%				−100.00%

^*^ p < 0.10, ^**^ p < 0.05, ^***^p < 0.01.

**Table 3:** Contributions to inequality in chronicity (n = 25,498)

	**Income**				**Deprivation**			
	**Partial effect**	**Elasticity**	**Contribution**	**% Contribution**	**Partial effect**	**Elasticity**	**Contribution**	**% Contribution**
*Age2*	0.056^***^	0.0535	0.0018	1.66%	0.057^***^	0.0543	0.0019	1.68%
*Age3*	0.131^***^	0.1435	0.0079	7.10%	0.134^***^	0.1475	0.0081	7.30%
*Age4*	0.215^***^	0.1455	−0.0146	−13.16%	0.225^***^	0.1525	−0.0153	−13.79%
*Age5*	0.297^***^	0.1089	−0.0273	−24.61%	0.312^***^	0.1143	−0.0286	−25.82%
*Female*	0.007	0.0171	−0.0004	−0.35%	0.007	0.0169	−0.0004	−0.34%
*Foreign*	−0.073^***^	−0.0169	0.0025	2.27%	−0.081	−0.0188	0.0028	2.52%
*Couple*	−0.023^***^	−0.0668	−0.0014	−1.27%	−0.015^*^	−0.0437	−0.0009	−0.83%
*Ed2*	−0.020^**^	−0.0208	0.0023	2.09%	−0.015^*^	−0.0163	0.0018	1.64%
*Ed3*	−0.025^**^	−0.0233	−0.0017	−1.50%	−0.011	−0.0102	−0.0007	−0.66%
*Ed4*	0.001	0.0001	0.0034	0.00%	0.016	0.0009	0.0000	0.04%
*Ed5*	−0.040^***^	−0.0445	−0.0154	−13.87%	−0.020^*^	−0.0217	−0.0075	−6.75%
*Unemployed*	0.079^***^	0.0251	−0.0063	−5.69%	0.062^***^	0.0196	−0.0049	−4.45%
*Student*	−0.004	−0.0013	0.0001	0.11%	−0.002	−0.0006	0.0001	0.05%
*Retired*	0.156^***^	0.0940	−0.0132	−11.87%	0.150^***^	0.0905	−0.0127	−11.42%
*Invalid*	0.747^***^	0.0624	−0.0101	−9.10%	0.742^***^	0.0620	−0.0100	−9.04%
*Home*	0.086^***^	0.0490	−0.0114	−10.26%	0.080^***^	0.0452	−0.0105	−9.46%
*Otherinact*	0.181^***^	0.0359	−0.0092	−8.27%	0.170^***^	0.0339	−0.0087	−7.80%
*Part-time*	0.038^**^	0.0100	−0.0001	−0.09%	0.032^*^	0.0084	−0.0001	−0.07%
*Freqfam2*	0.005	0.0057	0.0004	0.32%	0.007	0.0071	0.0004	0.40%
*Freqfam3*	0.022^**^	0.0154	−0.0009	−0.80%	0.018^*^	0.0122	−0.0007	−0.63%
*Freqfriend2*	−0.003	−0.0025	−0.0002	−0.15%	−0.004	−0.0037	−0.0002	−0.22%
*Freqfriend3*	0.051^***^	0.0291	−0.0050	−4.48%	0.045^***^	0.0254	−0.0043	−3.90%
*Contfam2*	−0.023^***^	−0.0222	0.0009	0.79%	−0.023^***^	−0.0223	0.0009	0.80%
*Contfam3*	0.009	0.0047	−0.0007	−0.66%	0.008	0.0040	−0.0006	−0.57%
*Contfriend2*	0.012^*^	0.0126	−0.0001	−0.08%	0.009	0.0092	−0.0001	−0.06%
*Contfriend3*	0.033^***^	0.0275	−0.0064	−5.78%	0.024^**^	0.0196	−0.0046	−4.11%
*Particip*	0.010	0.0291	0.0007	0.64%	0.009	0.0258	0.0006	0.57%
*Density2*	−0.002	−0.0023	0.0001	0.11%	−0.003	−0.0026	0.0001	0.13%
*Density3*	−0.033^***^	−0.0406	0.0067	6.00%	−0.032^***^	−0.0399	0.0066	5.91%
*Dephousing1*	0.032^***^	0.0361	−0.0032	−2.89%	0.019^**^	0.0219	−0.0019	−1.75%
*Dephousing2*	0.099^***^	0.0275	−0.0108	−9.73%	0.055^***^	0.0153	−0.0060	−5.42%
*Eqhincome*	0.006	0.2774	0.0100	9.04%	-	-	-	-
*Findepriv1*	-	-	-	-	0.044^***^	0.0390	−0.0036	−3.22%
*Findepriv2*	-	-	-	-	0.069^***^	0.0878	−0.0294	−26.51%
*Nodentist*	-	-	-	-	0.093^***^	0.0135	−0.0042	−3.74%
*Reg2*	0.065^***^	0.0083	0.0004	0.33%	0.079^***^	0.0101	0.0005	0.41%
*Reg3*	−0.035^*^	−0.0022	−0.0001	−0.09%	−0.028	−0.0018	−0.0001	−0.08%
*Reg4*	−0.023	−0.0050	−0.0009	−0.84%	−0.011	−0.0024	−0.0004	−0.40%
*Reg5*	−0.002	−0.0001	0.0000	−0.02%	0.015	0.0010	0.0003	0.24%
*Reg6*	0.020	0.0007	0.0000	−0.01%	0.030	0.0010	0.0000	−0.02%
*Reg7*	−0.018	−0.0026	−0.0002	−0.17%	−0.003	−0.0004	0.0000	−0.03%
*Reg8*	−0.075^***^	−0.0455	−0.0069	−6.22%	−0.067^***^	−0.0412	−0.0062	−5.63%
*Reg9*	0.025^*^	0.0071	−0.0008	−0.75%	0.031^**^	0.0088	−0.0010	−0.93%
*Reg10*	−0.057^***^	−0.0099	0.0012	1.08%	−0.051^***^	−0.0089	0.0011	0.97%
*Reg11*	−0.027^*^	−0.0030	0.0009	0.83%	−0.027^*^	−0.0030	0.0009	0.83%
*Reg12*	−0.045^***^	−0.0336	−0.0058	−5.22%	−0.037^***^	−0.0274	−0.0047	−4.26%
*Reg13*	−0.017	−0.0084	0.0002	0.19%	−0.017	−0.0083	0.0002	0.19%
*Reg14*	−0.017	−0.0015	−0.0002	−0.21%	−0.010	−0.0009	−0.0001	−0.12%
*Reg15*	−0.029^**^	−0.0226	0.0037	3.37%	−0.031^***^	−0.0239	0.0040	3.57%
*Reg16*	0.027	0.0036	−0.0003	−0.29%	0.030	0.0040	−0.0004	−0.32%
*Reg17*	−0.039	−0.0003	0.0000	0.03%	−0.038	−0.0003	0.0000	0.03%
*Reg18*	−0.050^*^	−0.0003	0.0000	−0.02%	−0.041	−0.0002	0.0000	−0.02%
*Reg19*	−0.044^***^	−0.0090	0.0015	1.33%	−0.050^***^	−0.0101	0.0017	1.49%
*Residual*			0.0013	1.15%			0.0260	23.60%
*Total*				−100.00%				−100.00%

^*^ p < 0.10, ^**^ p < 0.05, ^***^p < 0.01.

**Table 4:** Contributions to inequality in limiting conditions (n = 25,498)

	**Income**				**Deprivation**			
	**Partial effect**	**Elasticity**	**Contribution**	**% Contribution**	**Partial effect**	**Elasticity**	**Contribution**	**% Contribution**
*Age2*	0.034^***^	0.0347	0.0012	0.96%	0.034^***^	0.0348	0.0012	0.96%
*Age3*	0.095^***^	0.1120	0.0061	4.94%	0.099^***^	0.1158	0.0064	5.11%
*Age4*	0.142^***^	0.1034	−0.0104	−8.34%	0.152^***^	0.1108	−0.0111	−8.93%
*Age5*	0.255^***^	0.1002	−0.0251	−20.18%	0.271^***^	0.1063	−0.0266	−21.41%
*Female*	0.044^***^	0.1092	−0.0025	−1.97%	0.044^***^	0.1086	−0.0024	−1.96%
*Foreign*	0.004	0.0010	−0.0002	−0.12%	−0.009	−0.0021	0.0003	0.26%
*Couple*	−0.002	−0.0062	−0.0001	−0.10%	0.007	0.0210	0.0004	0.36%
*Ed2*	−0.022^***^	−0.0245	0.0027	2.20%	−0.017^**^	−0.0194	0.0022	1.74%
*Ed3*	−0.031^***^	−0.0318	−0.0023	−1.82%	−0.018^*^	−0.0178	−0.0013	−1.02%
*Ed4*	0.030	0.0018	0.0001	0.08%	0.049	0.0029	0.0002	0.13%
*Ed5*	−0.039^***^	−0.0459	−0.0159	−12.76%	−0.018^*^	−0.0208	−0.0072	−5.78%
*Unemployed*	0.064^***^	0.0218	−0.0055	−4.42%	0.047^***^	0.0159	−0.0040	−3.22%
*Student*	−0.047^***^	−0.0176	0.0017	1.38%	−0.044^***^	−0.0165	0.0016	1.30%
*Retired*	0.145^***^	0.0936	−0.0131	−10.54%	0.139^***^	0.0898	−0.0126	−10.11%
*Invalid*	0.678^***^	0.0607	−0.0098	−7.90%	0.668^***^	0.0598	−0.0097	−7.77%
*Home*	0.067^***^	0.0409	−0.0095	−7.63%	0.061^***^	0.0371	−0.0086	−6.92%
*Otherinact*	0.184^***^	0.0391	−0.0100	−8.04%	0.173^***^	0.0369	−0.0094	−7.59%
*Part-time*	0.017	0.0050	0.0000	−0.04%	0.011	0.0031	0.0000	−0.02%
*Freqfam2*	0.004	0.0048	0.0003	0.24%	0.006	0.0066	0.0004	0.33%
*Freqfam3*	−0.002	−0.0012	0.0001	0.06%	−0.007	−0.0050	0.0003	0.23%
*Freqfriend2*	0.022^**^	0.0223	0.0015	1.18%	0.020^**^	0.0207	0.0014	1.09%
*Freqfriend3*	0.073^***^	0.0446	−0.0076	−6.11%	0.066^***^	0.0401	−0.0068	−5.49%
*Contfam2*	−0.018^**^	−0.0191	0.0008	0.61%	−0.018^**^	−0.0188	0.0007	0.60%
*Contfam3*	0.012	0.0061	−0.0010	−0.77%	0.011	0.0056	−0.0009	−0.71%
*Contfriend2*	0.012	0.0132	−0.0001	−0.08%	0.008	0.0093	−0.0001	−0.05%
*Contfriend3*	0.038^***^	0.0334	−0.0078	−6.26%	0.027^***^	0.0239	−0.0056	−4.48%
*Particip*	0.015^**^	0.0481	0.0012	0.95%	0.014^**^	0.0453	0.0011	0.90%
*Density2*	0.019^**^	0.0190	−0.0010	−0.83%	0.020^**^	0.0193	−0.0011	−0.85%
*Density3*	−0.001	−0.0018	0.0003	0.24%	0.000	0.0003	−0.0001	−0.05%
*Dephousing1*	0.031^***^	0.0379	−0.0034	−2.71%	0.017^**^	0.0213	−0.0019	−1.52%
*Dephousing2*	0.119^***^	0.0353	−0.0139	−11.14%	0.069^***^	0.0205	−0.0081	−6.49%
*Eqhincome*	0.002	0.1122	0.0041	3.26%	-	-	-	-
*Findepriv1*	-	-	-	-	0.050^***^	0.0472	−0.0043	−3.47%
*Findepriv2*	-	-	-	-	0.079^***^	0.1080	−0.0362	−29.09%
*Nodentist*	-	-	-	-	0.104^***^	0.0162	−0.0050	−4.02%
*Reg2*	−0.034^**^	−0.0046	−0.0002	−0.17%	−0.022	−0.0029	−0.0001	−0.11%
*Reg3*	−0.082^***^	−0.0055	−0.0003	−0.21%	−0.077^***^	−0.0052	−0.0002	−0.20%
*Reg4*	−0.042^***^	−0.0096	−0.0018	−1.43%	−0.028^**^	−0.0065	−0.0012	−0.96%
*Reg5*	−0.047^***^	−0.0034	−0.0009	−0.70%	−0.032^**^	−0.0023	−0.0006	−0.48%
*Reg6*	−0.025	−0.0009	0.0000	0.02%	−0.014	−0.0244	−0.0001	0.01%
*Reg7*	−0.070^***^	−0.0108	−0.0008	−0.64%	−0.056^***^	−0.0087	−0.0006	−0.51%
*Reg8*	−0.090^***^	−0.0590	−0.0090	−7.20%	−0.083^***^	−0.0542	−0.0082	−6.60%
*Reg9*	−0.040^***^	−0.0120	0.0014	1.13%	−0.034^***^	−0.0102	0.0012	0.97%
*Reg10*	−0.051^***^	−0.0094	0.0011	0.92%	−0.044^***^	−0.0082	0.0010	0.80%
*Reg11*	−0.068^***^	−0.0080	0.0024	1.96%	−0.067^***^	−0.0079	0.0024	1.94%
*Reg12*	−0.048^***^	−0.0379	−0.0065	−5.26%	−0.039^***^	−0.0310	−0.0054	−4.30%
*Reg13*	−0.036^***^	−0.0195	0.0005	0.39%	−0.035^***^	−0.0190	0.0005	0.38%
*Reg14*	0.018	0.0018	0.0003	0.22%	0.026	0.0026	0.0004	0.32%
*Reg15*	−0.068^***^	−0.0555	0.0092	7.39%	−0.069^***^	−0.0565	0.0094	7.52%
*Reg16*	−0.021	−0.0030	0.0003	0.22%	−0.019	−0.0028	0.0002	0.20%
*Reg17*	−0.086^***^	−0.0006	0.0001	0.07%	−0.084^***^	−0.0006	0.0001	0.07%
*Reg18*	−0.045^*^	−0.0003	0.0000	−0.02%	−0.034	−0.0002	0.0000	−0.01%
*Reg19*	−0.065^***^	−0.0141	0.0023	1.86%	−0.070^***^	−0.0152	0.0025	2.01%
*Residual*			−0.0035	−2.89%			0.0208	16.93%
*Total*				−100.00%				−100.00%

^*^ p < 0.10, ^**^ p < 0.05, ^***^p < 0.01.

Results from Tables 2, 3, 4 show that the probability of declaring health problems (irrespective of the ill-health variable used) grows as individuals get older and depends positively and significantly on labour inactivity (except for students) and on housing and financial deprivation. The absence of dental treatment due to financial problems also has a significant impact on ill-health in those models including this regressor. Income, however, is not significant. Education, on the contrary, reduces the probability of declaring fair, poor or very poor health, and also the probability of suffering from chronic or limiting conditions. A set of regressors appear to be statistically significant only in some of the estimated models. Thus, being female is significantly and positively associated with ill-health for models referring to self-assessed health (SAH) and limiting conditions; and being a foreigner, living in a couple or in a sparsely populated area tend to reduce the presence of chronic conditions. With respect to social interactions, all the models show that having a poor frequency of contacts with friends increases the probability of declaring health problems. However, the impact of family contacts on health is not as clear. Regional dummies also show a significant impact on most models, indicating that individuals living in Galicia (the reference category) are, in general, more prone to declare ill-health problems compared to other Spaniards. However, this result may be reflecting cultural effects rather than pure inequality in health.

Variations in elasticities are responsible for the observed changes in the contribution of variables to inequality across models, given that the concentration index remains constant. As shown in Tables 2, 3, 4, health inequality favouring the better-off is mainly explained, besides age, by social factors such as labour status or financial deprivation, depending on the estimated models. Education and housing deprivation also play an important role in inequality favouring the better-off, followed by variables representing social interactions. Table 5 shows the relative contribution to inequality of several groups of factors. Demographic determinants, including sex and age, explain from 24.59% to 32.21% of total inequality. Activity status also shows a very high contribution to inequalities in health favouring the better-off. It explains approximately 20% of unequal distribution of self-assessed health, and more than 40% of inequality in the distribution of chronicity. Moreover, the contribution of education ranges from 5% to more than 18%. For those models including financial deprivation, this is the most important determinant of inequality, which contributes from 32.46% to 36.58% to the unequal distribution of ill-health favoring the better-off. These results are consistent with those obtained by Hernández-Quevedo et al. [12], who analyze the contribution of health determinants to inequality in the distribution of health limitations for the year 2007.

**Table 5:** Relative contributions to inequality (summary)

	**Models including income**				**Models including financial deprivation**		
	**SAH**	**Chronic**	**Limit**		**SAH**	**Chronic**	**Limit**
**Demography**	−31,05%	−29,36%	−24,59%	**Demography**	−32,21%	−30,97%	−26,23%
**Education**	−18,27%	−13,27%	−12,30%	**Education**	−12,91%	−5,72%	−4,93%
**Activity status**	−21,51%	−45,17%	−37,18%	**Activity status**	−20,00%	−42,20%	−34,34%
**Social interactions**	−8,21%	−10,19%	−10,18%	**Social interactions**	−6,16%	−7,73%	−7,59%
**Housing deprivation**	−13,31%	−12,62%	−13,85%	**Housing deprivation**	−8,56%	−7,17%	−8,01%
**Income**	−6,56%	9,04%	3,26%	**Financial deprivation**	−32,46%	−33,47%	−36,58%
**Place of residence**	−3,04%	−0,59%	−2,03%	**Place of residence**	−1,49%	1,97%	0,13%
**Other**	−0,96%	1,00%	−0,23%	**Other**	−0,25%	1,69%	0,61%
**Residual**	2,91%	1,15%	−2,89%	**Residual**	14,04%	23,60%	16,93%

The results also show that housing deprivation, one of the components of social exclusion, explains the socioeconomic inequalities in health observed for Spaniards from 7.17% to 13.85%. Its contribution to pro-rich inequality results from the combination of positive elasticities and negative concentration indices and is mainly determined by the effect of the variable that indicates the presence of two or more deprivation problems.

Relevant contributions from social interactions correspond only to those dummies indicating a very scarce frequency of contacts with friends. Ill-health elasticity is positive for these two variables in all the estimated models, thus indicating that social isolation tends to favour health problems (or, more accurately, the perception of health problems). Furthermore, the negative concentration indices of both dummies show that isolation is relatively concentrated on poor people, while more frequent contacts with friends are relatively concentrated among the rich (see Table 1). Again, as resulting from the combination of positive elasticities and negative concentration indices, social interactions contributes to pro-rich inequality in a percentage ranging from 6.16% to 10.19%.

Place of residence (grouping variables about density and region) and other factors (including *Foreign* and *Couple*) have minor relevance in the explanation of inequality. The small impact of the *Foreign* dummy may be linked to the sub-representation of migrants in the sample analyzed. Although foreigners represent 5% of the total sample according to Table 1, the percentage of adult migrants over adult population living in Spain in 2006 reached 11% [42]. Moreover, it may also be related to the so-called ‘healthy-migrant effect’, which implies that only those individuals with good health migrate.

Finally, the residuals play a very different role across models. The magnitude of the contribution of residuals in some of the models is linked to the fact that income is not included as a regressor. The error term of equation (9) is a measure of the covariance between the residuals of the regression model and the position that each individual occupies in the income distribution. Thus, the unexplained part of the model should be near zero if income appears as an explanatory variable [43]. Compared to the models where income is included as a regressor, those including financial deprivation show a lower contribution of education, housing and social interactions to health inequality.

The contribution of some groups of determinants also differs depending on the ill-health variable used. Particularly, the contribution of education is significantly more relevant when self-assessed health is analyzed, and it is reduced when more objective ill-health measures are employed. Conversely, the contribution of activity status is much higher in those models where declaring chronic or limiting conditions are estimated. However, both education and activity status contribute to inequality favoring the better-off, independently of the observed model. This is not the case with income, which only in the SAH model seems to contribute to pro-rich inequality. These results may confirm that self-assessed health may be systematically correlated with some characteristics of individuals, such as income level or education, as was mentioned in the previous section.

This study has a number of limitations. First, it is a cross-sectional study, so it is not possible to discuss its findings in terms of causal relationships. Second, the concentration index is not neutral and assumes specific parameters on inequality aversion [34]. A third group of limitations has to do with the definition of ill-health variables. The indicators employed are not based on clinical reports, which may imply that they are subject to bias depending on personal or socioeconomic characteristics. Furthermore, this bias may depend on the ill-health indicator used. Moreover, the conversion of two original multi-categorical variables (self-assessed health and limitations in daily activities) into dummies also may introduce some distortion. Finally, some problems may affect the estimation results. Several relevant determinants of health, such as lifestyle, are excluded from the regressions, as they are not collected by the EU-SILC. Also, the variable ‘*Nodentist*’, as it reflects unmet need, could be considered as endogenous and hence cause some bias in the estimation. However, when this variable is excluded from the analysis the results hardly change. Additionally, some regressors are significantly correlated and may have causal relations between them. This is the case between the proxies of housing deprivation and income, and also between housing deprivation and financial deprivation, although rank correlations never exceed 0.36.

## Conclusions

From the previous analysis some conclusions may be drawn. Although mean health levels in Spain are high, socioeconomic inequalities in health are statistically significant. This is consistent with previous evidence which also shows that, when compared to other European countries, Spain occupies an intermediate position in the ranking of social inequalities in health [6,12]. Labour status, education and other socioeconomic conditions, which have been proved to be influential independently on the evaluated dimensions of ill-health, are the factors that mainly explain health inequalities in Spain. Therefore, the relevance of social determinants is confirmed and also the fact that inequalities can be mostly reduced or shaped by policy. This paper also highlights the contribution of additional factors on health inequalities, as housing conditions and the absence of friend networks, which are also pieces of the social integration process. The major role played on health inequality by variables taking part in social exclusion points to the need to focus on the most vulnerable groups.

Moreover, the results in this paper highlight the relevance of designing broad policies aimed at reducing health inequalities instead of concentrating efforts only on health care. Therefore, the results are consistent with the design of the strategy ‘Health in All Policies’, which has been promoted by the World Health Organization and adopted in some countries. This strategy calls for integrating the health dimension into areas such as education, employment, housing, transport and environmental and fiscal policies [44]. The Public Health Act recently approved by the Spanish Parliament reflects this spirit and states that ‘*policies, plans and programs affecting the health of the population will promote the reduction of social inequalities in health and will include measures regarding their social determinants, including relevant specific objectives*’ (Art. 3) [21]. However, the present economic context may jeopardize these intentions. Recent reforms aimed at reducing the public deficit such as wage cuts for civil servants and cuts in social benefits may deepen the effects of a serious economic crisis that already has resulted in a growth of unemployment, poverty and social exclusion [45,46]. Besides, most recent reforms implemented in the NHS imply the exclusion of public coverage for different groups of population –among them, migrants in an irregular situation-, the restriction of covered benefits and the increase of copayments [47]. Further research will be needed to measure the real impact of these reforms on health and the health gradient in Spain.

## Endnote

^a^As a weight is assigned to every observation from the EU-SILC, the variable r_i_ in equation (3) has to be computed with the following expression: $r_{i}=\sum_{j=0}^{i=1}w_{j}+\frac{w_{i}}{2}$, where *w*_*i*_ is the sample weight scaled to sum 1, the observations are ranked from the lowest to the highest income, and *w*_*0*_*=0*[36].

## Competing interests

The autor declares that they have no competing interests.

## Acknowledgments

I am grateful to the Spanish Institute for Fiscal Studies for its financial support. I also would like to thank Angela Blanco for her help in the development of this research. The usual disclaimer applies.
